# Rate–Distortion–Perception Trade-Off in Information Theory, Generative Models, and Intelligent Communications

**DOI:** 10.3390/e27040373

**Published:** 2025-03-31

**Authors:** Xueyan Niu, Bo Bai, Nian Guo, Weixi Zhang, Wei Han

**Affiliations:** Theory Lab, 2012 Labs, Huawei Technologies Co., Ltd., No. 3 Xinxi Rd., Beijing 100085, China; baibo8@huawei.com (B.B.); guonian4@huawei.com (N.G.); zhangweixi1@huawei.com (W.Z.); harvey.hanwei@huawei.com (W.H.)

**Keywords:** rate–distortion–perception trade-off, perceptual fidelity, lossy compression, AI-empowered communication

## Abstract

Traditional rate–distortion (RD) theory examines the trade-off between the average length of the compressed representation of a source and the additive distortions of its reconstruction. The rate–distortion–perception (RDP) framework, which integrates the perceptual dimension into the RD paradigm, has garnered significant attention due to recent advancements in machine learning, where perceptual fidelity is assessed by the divergence between input and reconstruction distributions. In communication systems where downstream tasks involve generative modeling, high perceptual fidelity is essential, despite distortion constraints. However, while zero distortion implies perfect realism, the converse is not true, highlighting an imbalance in the significance of distortion and perceptual constraints. This article clarifies that incorporating perceptual constraints does not decrease the necessary rate; instead, under certain conditions, additional rate is required, even with the aid of common and private randomness, which are key elements in generative models. Consequently, we project an increase in expected traffic in intelligent communication networks with the consideration of perceptual quality. Nevertheless, a modest increase in rate can enable generative models to significantly enhance the perceptual quality of reconstructions. By exploring the synergies between generative modeling and communication through the lens of information-theoretic results, this article demonstrates the benefits of intelligent communication systems and advocates for the application of the RDP framework in advancing compression and semantic communication research.

## 1. Introduction

**Figure 1:**
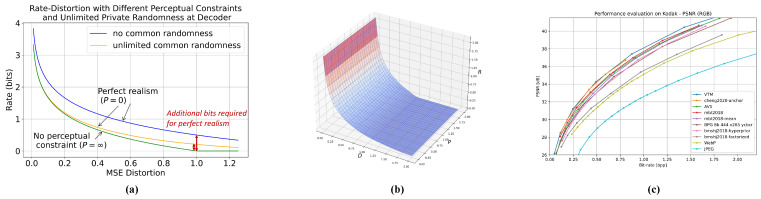
Rate–distortion–perception trade-off. (**a**) Rate–distortion curves with and without the realism constraint for a Gaussian source. The perceptual constraint slightly increases the bitrate required in theory, suggesting an increase in traffic in communication networks. (**b**) Information rate–distortion–perception frontier for a Gaussian source with MSE distortion and 2-Wasserstein perceptual constraint. (**c**) Rate–distortion performance evaluation of various practical image compression algorithms using the PSNR distortion metric. The evaluation is conducted with the CompressAI package [14], covering a range of methods from the standard JPEG to SoTA learning-based compression techniques such as [15].

For decades, rate–distortion (RD) theory has served as the de facto standard for evaluating real-world applications involving compression and communication. With the advances in artificial intelligence technologies, the dimension of perceptual fidelity has been increasingly recognized as a crucial aspect. While traditional distortion measures like mean squared error (MSE) have served well in many applications, they fail to capture the perceptual quality that humans actually experience [1,2,3]. This disconnect has motivated the integration of perceptual measures into RD, leading to the emergence of the rate–distortion–perception (RDP) framework [4]. The information RDP function incorporates an additional perceptual constraint measured by the divergence between the input and reconstruction distributions within the context of lossy compression using machine learning. Unlike conventional distortion measures, perceptual fidelity captures the global properties of the entire coding block rather than focusing on individual sample positions, giving rise to new coding theorems. Consequently, RD plots, which have long been used to evaluate practical source coding algorithms, should be updated to incorporate this new dimension in alignment with the emerging coding theorems (see Figure 1b,c for a comparison of the RDP frontier with the RD curves). This development has also inspired a variety of innovative source coding methods that rely on generative models [5,6,7,8,9,10,11]. As emerging techniques are increasingly applied to lossy source coding and communication systems in general, the RDP trade-off offers a novel perspective for evaluating the performance of machine-learning-aided systems. Recent advances in AI-empowered communication have reimagined communication systems as a joint optimization of transmitter and receiver through end-to-end training for reconstruction tasks [12,13]. Therefore, in communication systems where downstream tasks demand high perceptual fidelity, such as interactive AI agents and the reconstruction of visually pleasing content, the RDP framework is particularly useful. This paper explores the critical role of perceptual constraints in information theory and their impact on practical generative modeling scenarios, which drives the development of a wide range of compression and communication algorithms that integrate various generative model architectures with end-to-end training objectives. We demonstrate how incorporating perceptual constraints into the traditional RD framework offers a comprehensive perspective for evaluating the performance of AI-empowered systems in communication and potentially increases network traffic.

The capacity to perceive, communicate, and learn from the environment, as well as to construct mental models that internally simulate potential outcomes, is fundamental to both human and artificial intelligence. In early neuroscience research, perception was conceptualized as a form of hypothesis testing [16], a notion that naturally aligns with the divergence measure for perceptual quality in information theory. More recently, the theory of predictive coding has posited that generative models of the world are derived from sensory input, perception being understood as the minimization of prediction errors [17,18]. Developing models capable of mimicking human-like perception and reasoning, including visual aesthetics and consistency; language perplexity and fluency, among others, is essential for the creation of useful generative models. Contemporary large language models (LLMs) and multi-modal models have demonstrated remarkable capabilities, such as memorization [19], generation of images and videos based on descriptive prompts [20], retrieval and reasoning [21]. During the training of these generative models, the loss function typically combines both distortion-oriented MSE and perception-oriented cross-entropy losses, weighted to balance distortion and perception constraints (see Equation (21)). For downstream tasks such as image synthesis, a key challenge is to balance the faithfulness to user inputs and the realism of the generated outputs [22]. Within the RDP framework, the perceptual quality of reconstruction is assessed by the similarity between the distributions of the input and the reconstruction, with the encoder and decoder potentially aided by generative models. This alignment naturally supports the objectives of generative modeling. Thus, in contemporary intelligent compression and communication systems, the quality of reconstruction should be evaluated based on both the perceptions of end users, whether human or machine, and the average distortions.

The perception axis plays a pivotal role in both source coding and communication systems in general. In information theory, the source coding problem aims to minimize the amount of information needed to transmit a message by eliminating redundancy, thereby achieving effective compression. To assess the performance, a certain level of reconstruction error is permissible. For instance, when compressing and subsequently recovering a bit string using a decoder, the Hamming distance can be used to quantify the number of errors, thereby providing a measure of distortion. Conventional RD theory, originally developed by Claude Shannon, has effectively characterized the minimal rate required to satisfy distortion constraints. For stationary sources, it is well established that deterministic encoders and decoders can achieve asymptotically optimal RD performance [23]. However, the introduction of perceptual constraints suggests that deterministic coding may no longer be optimal [24]. Randomization has been demonstrated to be essential for achieving realism constraints through stochastic coding [25,26,27]. The requirement for the output to conform to a specified distribution is known as the realism constraint [28,29,30,31]. The zero-distortion constraint corresponds to the well-known zero-error source coding problem [23], which also implies perfect realism. However, perfect realism, which requires the distributions of the source and reconstruction to be identical, does not necessarily imply zero distortion. Nevertheless, perceptual constraints restrict the reconstruction process, thereby increasing the required rate. For example, consider a pangram—a sentence using each letter of the English alphabet with balanced letter counts. If a decoder is required to reconstruct a sentence while adhering to a specific letter distribution, this scenario exemplifies the perfect realism condition. A “perfect pangram” is extremely rare due to the overuse of common letters and the scarcity of rare ones; for instance, the classic pangram “The quick brown fox jumps over a lazy dog” contains repeated Es and Os. This suggests that perceptual constraints are closely related to semantics and can be highly restrictive, thereby carrying significant amounts of information.

In practical communication systems, the idealized assumptions underlying the separation theorem, upon which modern communication systems are founded, are rarely met. The separation theorem posits that separate source and channel coding (SSCC) is theoretically optimal in terms of distortion, with source coding eliminating data redundancy and channel coding ensuring reliable transmission in noisy and interference-prone environments. Moreover, under certain conditions, incorporating perceptual constraints renders separation suboptimal in the absence of common randomness between the encoder and decoder [32]. Recent research has increasingly focused on AI-empowered communication systems, where communication nodes are enhanced through machine learning techniques, such as generative modeling, to improve the comprehension and generation of high-fidelity content for downstream tasks, including image and video transmission, augmented and virtual reality (AR/VR), the Internet of Things (IoT), and vehicular-to-everything (V2X) applications. These systems are trained end-to-end for downstream tasks using generative objectives that inherently incorporate perceptual measures. Inspired by these information-theoretic results, which suggest that common and private randomness at the encoder and decoder provide advantages in terms of the RDP trade-off, AI-empowered generative models can be deployed at the transmitter and receiver nodes, with these randomness sources serving as shared or private random seeds.

As the main purpose of this paper, we are devoted to highlighting efforts in the field of information theory aimed at elucidating the fundamental limits of source coding and communication with perceptual-oriented metrics. Inspired by these findings, we demonstrate the utility of common and private randomness in generative modeling architectures, such as variational auto-encoders (VAEs), generative adversarial networks (GANs), diffusion models, and transformers, through the lens of the RDP trade-off. These generative modeling architectures can be designed and deployed at communication nodes to support downstream tasks requiring high perceptual fidelity. We provide a comprehensive overview of common perceptual measures in Section 2. Subsequently, Section 3 discusses the information-theoretic results and their implications with respect to the additional rate required to meet the new perceptual constraint. Section 4 introduces several generative modeling architectures, including VAEs, GANs, diffusion models, and transformers, used in communication systems, emphasizing the importance of high perceptual fidelity in downstream machine learning tasks. Section 5 characterizes the role of generative models in AI-empowered semantic communication systems. Finally, Section 6 explores future directions in information theory and communication. Through the lens of the RDP trade-off, we demonstrate that the introduced perceptual constraint leads to an increase in expected traffic within communication networks. The key takeaways include the following:

- Perceptual constraints fundamentally alter the rate–distortion trade-off, requiring additional rate even with unlimited common randomness.
- Generative models naturally align with RDP objectives, making them powerful tools for developing perceptually optimized communication systems.
- The practical implementation of RDP-aware systems faces challenges related to computational complexity and the need for robustness across diverse channel conditions.

### Notations

We use capital letters such as *X* to denote random variables, lowercase letters such as *x* to denote their corresponding instances, and calligraphic letters such as X to denote sets of alphabets. Let Prob(X) denote the set of probability measures defined on X, and let $P_{X}\left(x\right),P_{XY}\left(x,y\right)$, and $P_{Y|X}\left(y|x\right)$ denote the probability distribution of *X*, the joint distribution, and the conditional distribution of *Y* given X=x, respectively. We omit the arguments of the distributions when doing so does not cause ambiguity. The *n*-sequence $\left(X_{1},X_{2},\ldots ,X_{n}\right)$ is denoted by $X^{n}$. Suppose that ${\left\{X_{i}\right\}}_{i=1}^{\infty }$ and ${\left\{Y_{i}\right\}}_{i=1}^{\infty }$ are memoryless sources; then, $P_{X^{n}Y^{n}}=\prod_{i=1}^{n}P_{XY}={⊗}_{i=1}^{n}P_{X_{i}Y_{i}}$. We use [m] to denote the set {1,2,…,⌊m⌋} for m>0, and we use C to denote the codebook. For sequences $\left(x^{n},y^{n}\right)\in X^{n}\times Y^{n}$, the empirical distribution is defined as

(1) $${\hat{P}}_{x^{n},y^{n}}\left(x,y\right)=\frac{1}{n}\sum_{i=1}^{n}1\left\{\left(x_{i},y_{i}\right)=\left(x,y\right)\right\}.$$

Extensions to multiple arguments can be defined similarly. Throughout the paper, we use $\Delta \left(\cdot ,\cdot \right):X^{n}\times X^{n}\mapsto R$ to denote an additive distortion measure, and we use the calligraphic letter $D\left(\cdot ,\cdot \right):\mathrm{Prob}\left(X^{n}\right)\times \mathrm{Prob}\left(X^{n}\right)\mapsto R$ to denote the measure of perceptual fidelity. We define ${\bar{R}}_{\geq 0}:=R_{\geq 0}\cup \left\{+\infty \right\}$.

## 2. Distortion and Perceptual Measures

We set out to explore the interplay between conventional distortion measures and the recently introduced perceptual measures. Details regarding the emerging class of perceptual measures, including the total variation (TV) distance, the Kullback–Leibler (KL) divergence, and the Wasserstein distance, are provided. Additionally, we present a set of assumptions and a series of distance–divergence inequalities. These elements highlight the fundamental differences and connections between distortion and perceptual measures, thereby motivating the information-theoretic results. We demonstrate that neither the distortion measure nor any of the perceptual measures, including the TV distance, KL divergence, and Wasserstein distance, dominate each other. Instead, they synergistically contribute to the RDP trade-off.

### 2.1. Distortion Measures

In the conventional additive distortion setting, the distortion measure is defined as the average of a per-letter distance.

**Definition 1.**  *Given a per-letter distortion measure $\delta :X\times X\mapsto [0,d_{\max}]$ with $d_{\max}<\infty$, the (average) distortion between two sequences $x^{n}$ and $y^{n}$ is defined as*

$$\Delta \left(x^{n},y^{n}\right):=\frac{1}{n}\sum_{i=1}^{n}\delta \left(x_{i},y_{i}\right).$$

*Here, we assume that the distortion is upper-bounded by $d_{\max}$.*

This bounded distortion enables the derivation of a bound relating the distortion measure to the perceptual measure, as shown in Equation (11), which will be introduced below.

### 2.2. Perceptual Measures in Information Theory

To quantify the proximity between the reconstruction distribution and another probability distribution, several perceptual measures are widely studied in information theory. These include the total variation (TV) distance, the Kullback–Leibler (KL) divergence, and the Wasserstein distance.

#### 2.2.1. Total Variation Distance

The TV distance defined in the following is widely used in information theory. Given two distributions $P_{X}$ and $P_{Y}$ defined on the same σ-algebra (X,F), the TV distance is defined as

$$D_{\mathrm{TV}}\left(P_{X},P_{Y}\right)=\underset{S\subseteq X}{\sup}\left|P_{X}\left(S\right)-P_{Y}\left(S\right)\right|.$$

The TV distance is equivalent to the $L_{1}$ norm when the alphabets are finite, making it a natural choice in the Lebesgue space, i.e., when $P_{X}$ and $P_{Y}$ are probability mass functions,

(2) $$D_{\mathrm{TV}}\left(P_{X},P_{Y}\right)=\frac{1}{2}∥P_{X}-P_{Y}{∥}_{1}=\frac{1}{2}\sum_{x\in X}\left|P_{X}\left(x\right)-P_{Y}\left(x\right)\right|.$$

From Equation (2) and the triangle inequality for real numbers, we have the triangle inequality

$$D_{\mathrm{TV}}\left(P_{X},P_{Y}\right)\leq D_{\mathrm{TV}}\left(P_{X},P_{Z}\right)+D_{\mathrm{TV}}\left(P_{Z},P_{Y}\right).$$

This implies that the TV distance does not tensorize. In fact, we have the following folklore knowledge:

(3) $$D_{\mathrm{TV}}\left(P_{X_{1}X_{2}},P_{Y_{1}Y_{2}}\right)\leq D_{\mathrm{TV}}\left(P_{X_{1}},P_{Y_{1}}\right)+D_{\mathrm{TV}}\left(P_{X_{2}},P_{Y_{2}}\right)-D_{\mathrm{TV}}\left(P_{X_{1}},P_{Y_{1}}\right)\cdot D_{\mathrm{TV}}\left(P_{X_{2}},P_{Y_{2}}\right).$$

#### 2.2.2. KL Divergence

Another widely used divergence measure in information theory is the KL divergence. Given two distributions $P_{X}$ and $P_{Y}$ defined on the same σ-algebra (X,F), when X is finite,

$$D_{\mathrm{KL}}\left(P_{X}∥P_{Y}\right)=\sum_{x\in X}P_{X}\left(x\right)\log\frac{P_{X}\left(x\right)}{P_{Y}\left(x\right)}.$$

When $P_{X}$ and $P_{Y}$ are probability density functions, the KL divergence is defined as

$$D_{\mathrm{KL}}\left(P_{X}∥P_{Y}\right)=\int_{x\in X}P_{X}\left(x\right)\log\frac{P_{X}\left(x\right)}{P_{Y}\left(x\right)}\;dx,$$

and, by convention, $D_{\mathrm{KL}}\left(P_{X}∥P_{Y}\right)=\infty$ when $P_{X}$ is not absolutely continuous with regard to $P_{Y}$. The KL divergence is not a distance metric because it is asymmetric between the two arguments and does not satisfy the triangle inequality.

#### 2.2.3. Wasserstein Distance

The Wasserstein distance, rooted in the theory of optimal transport, is a powerful tool with broad applications in deep learning as a perceptual measure [33]. It has also garnered significant attention in information theory, particularly following the pioneering work of Marton on distance–divergence inequalities [34,35].

**Definition 2.**  *Given p≥1 and a Polish space (X,c), the $L_{p}$-Wasserstein distance is defined as*

$$W_{p}\left(P_{X},P_{Y}\right)=\inf\;{\left(E\left[c^{p}\left(X,Y\right)\right]\right)}^{\frac{1}{p}},$$

*where the infimum is over all jointly distributed random variables (X,Y)∈Prob(X×X).*

For each p≥1, $W_{p}\left(,\;\right)$ defines a metric on Prob(X). In practice, $W_{1}$ and $W_{2}$ are most widely used.

#### 2.2.4. Common Assumptions

In the more general case, several assumptions are made regarding perceptual measures across different settings, which we briefly discuss here.

**Assumption 1**  (Non-negativity). *For arbitrary distributions $P_{X}$ and $P_{Y}$ defined on the σ-algebra (X,F), the perceptual measure satisfies*

$$D\left(P_{X},P_{Y}\right)\geq 0\;\;\mathrm{and}\;\;D\left(P_{X},P_{Y}\right)=0\;\;\mathrm{if}\;\mathrm{and}\;\mathrm{only}\;\mathrm{if}\;\;P_{X}\overset{d}{=}P_{Y}.$$

**Assumption 2**  (Convexity in the second argument). *For $\lambda_{1},\lambda_{2},\ldots ,\lambda_{n}\in R_{\geq 0}$ such that $\sum_{i}\lambda_{i}=1$,*

(4) $$D\left(P,\sum_{i=1}^{n}\lambda_{i}Q_{i}\right)\leq \sum_{i=1}^{n}\lambda_{i}D_{\mathrm{TV}}\left(P,Q_{i}\right).$$

This convexity is a property satisfied by the *f*-divergence family, including the TV distance and the KL divergence, as well as the 1-Wasserstein distance.

Some members of the *f*-divergence family, such as the Hellinger distance and the $\chi^{2}$-divergence, exhibit tensorization. This means that, for product distributions, these divergences can be easily expressed in terms of the marginal distributions. Tensorization, or additivity under products, is crucial for establishing converse proofs.

**Assumption 3**  (Tensorization).

(5) $$D\left({⊗}_{i=1}^{n}P_{X_{i}},P_{Y^{n}}\right)\geq \sum_{i=1}^{n}D\left(P_{X_{i}},P_{Y_{i}}\right).$$

Notably, the KL divergence and $W_{2}$ distance are tensorizable, in the sense that

(6) $$\begin{array}{cc} D_{\mathrm{KL}}\left({⊗}_{i=1}^{n}P_{X^{i}}∥{⊗}_{i=1}^{n}P_{Y^{n}}\right) & =\sum_{i=1}^{n}D_{\mathrm{KL}}\left(P_{X_{i}}∥P_{Y_{i}}\right), \end{array}$$

(7) $$\begin{array}{cc} W_{2}\left({⊗}_{i=1}^{n}P_{X^{i}}∥{⊗}_{i=1}^{n}P_{Y^{n}}\right) & =\sum_{i=1}^{n}W_{2}\left(P_{X_{i}}∥P_{Y_{i}}\right). \end{array}$$

The TV distance, however, is not tensorizable.

We may also consider a sequence of perceptual measures to analyze performance over coding blocks. The sub-decomposable assumption introduced in [32] ensures that coding over longer sequences is advantageous.

**Assumption 4**  (Sub-decomposability). *A sequence of perception measures $D_{n}\left(\cdot ,\cdot \right):\mathrm{Prob}\left(X^{n}\right)\times \mathrm{Prob}\left(X^{n}\right)\mapsto R$ is called sub-decomposable if*

(8) $$D_{n}\left({⊗}_{i=1}^{k}P_{X_{l_{i-1}+1}^{l_{i}}},{⊗}_{i=1}^{k}P_{X_{l_{i-1}+1}^{l_{i}}}\right)\leq \sum_{i=1}^{k}D_{n_{i}}\left(P_{X_{l_{i-1}+1}^{l_{i}}},P_{Y_{l_{i-1}+1}^{l_{i}}}\right)$$

*for any positive integers $n_{1},n_{2},\ldots ,n_{k}$ such that $\sum_{i=1}^{k}n_{i}=n$, where $l_{i}:=\sum_{j=1}^{i}n_{i}$ with $l_{0}:=0$.*

By definition, any tensorizable measure, such as the KL divergence, is also sub-decomposable. The non-tensorizable TV distance is sub-decomposable as well, as shown in Equation (3).

### 2.3. Interplay Between Distortion and Perception Constraints

Distortion and perception measures, though distinct, are closely intertwined through a series of distance–divergence inequalities. For instance, in the product space $X^{n}$, the transportation–cost inequalities pioneered by Marton [34] can be employed to derive concentration results.

A notable bound relates the expected values of bounded functions to the TV distance:

(9) $$|E_{P_{X}}\left[f\left(X\right)\right]-E_{P_{Y}}\left[f\left(X\right)\right]|\leq \underset{x}{\sup}\left|f\left(x\right)\right|\;D_{\mathrm{TV}}\left(P_{X},P_{Y}\right),$$

which facilitates the analysis of expected values using the TV distance. This relationship is particularly useful in machine learning scenarios where the objective is to minimize loss functions that depend on expected values. In such cases, the optimization problem can be reformulated as minimizing the TV distance. Specifically, given that the distortion is bounded by $d_{\max}$, and assuming that $P_{X^{n}Y^{n}}$ and $Q_{X^{n}Y^{n}}$ are i.i.d. distributions, we have the following:

(10) $$|E_{P}\left[D\left(X^{n},Y^{n}\right)\right]-E_{Q}\left[D\left(X^{n},Y^{n}\right)\right]]|\leq d_{\max}D_{\mathrm{TV}}\left(P,Q\right).$$

Similarly, the bounded distortion measure exhibits a property elucidated in Lemma 5 of [36] with respect to the TV distance: if $P_{XY},Q_{XY}$ are probability mass functions with $E_{P_{XY}}\left[\delta \left(X,Y\right)\right]=A$ and $D_{\mathrm{TV}}\left(P_{XY},Q_{XY}\right)<\epsilon$, then

(11) $$E_{Q_{XY}}\left[\delta \left(X,Y\right)\right]\leq A+\epsilon d_{\max}.$$

Perceptual measures can also be related through a set of distance–divergence inequalities. A fundamental relationship between the KL divergence and the TV distance, originally due to Pinsker [37], states the following:

(12) $$D_{\mathrm{TV}}\left(P_{X},P_{Y}\right)\leq \sqrt{\frac{1}{2}D_{\mathrm{KL}}\left(P_{X},P_{Y}\right)}.$$

For a finite alphabet, the reverse Pinsker inequality is available (see Lemma 6.3 in [38]):

(13) $$D_{\mathrm{KL}}\left(P_{X},P_{Y}\right)\leq \frac{D_{\mathrm{TV}}^{2}\left(P_{X},P_{Y}\right)}{\min_{y\in X}P_{Y}\left(y\right)}.$$

Moreover, for i.i.d. distributions $P_{Y^{n}}$ and $P_{X^{n}}\ll P_{Y^{n}}$, we have (see Equation (30) in [39])

(14) $$D_{\mathrm{KL}}\left(P_{X^{n}},P_{Y^{n}}\right)=O\left(\left(n-\log\left(D_{\mathrm{TV}}\left(P_{X^{n}},P_{Y^{n}}\right)\right)\right)D_{\mathrm{TV}}\left(P_{X^{n}},P_{Y^{n}}\right)\right).$$

Assuming that the diameter of X is bounded by *B*, we also have (see Theorem 6.15 in [40]) the following:

(15) $$W_{1}\left(P_{X},P_{Y}\right)\leq B\cdot D_{\mathrm{TV}}\left(P_{X},P_{Y}\right).$$

For coding blocks on $\mathrm{Prob}(X^{n})$, Wasserstein distances in product spaces can be naturally related to the distortion measure:

$$\Delta_{\beta ,n}\left(P_{X^{n}},P_{Y^{n}}\right):=\underset{\pi \in \Pi (P_{X^{n}},P_{Y^{n}})}{\inf}\;{\left(E_{\pi }\left[\sum_{i=1}^{n}\delta^{p}\left(X_{i},Y_{i}\right)\right]\right)}^{\frac{1}{p}},$$

and, according to [35], under certain conditions,

(16) $$W_{1,n}\left(P_{X^{n}},P_{Y^{n}}\right)\leq \mathrm{Const}.\sqrt{\frac{1}{2}D_{\mathrm{KL}}\left(P_{X^{n}},P_{Y^{n}}\right)}.$$

These relationships connect common perceptual measures to the TV distance, which is frequently considered in information-theoretic settings. The perceptual measures discussed—TV distance, KL divergence, and Wasserstein distance—each offer unique advantages depending on the application context. TV distance provides a straightforward measure of distributional similarity, KL divergence captures directional divergence between distributions, and Wasserstein distance offers a geometrically intuitive measure of the transport cost between probability distributions.

## 3. Information-Theoretic Results

Having established the theoretical foundations of perceptual measures, we now turn to their implications in information theory. Understanding how these measures interact with rate and distortion constraints requires a rigorous information-theoretic analysis of the RDP trade-off. This section summarizes recent information-theoretic results for the RDP problem. Typically, a set of distortion constraints **(D)** and a set of perceptual constraints **(P)** are enforced to ensure that the distortion and perceptual fidelities are bounded by given constants *D* and *P*, respectively.

As in classical information theory, we consider a memoryless source ${\left\{X_{i}\right\}}_{i=1}^{\infty }$ drawn from finite alphabets X. For continuous sources, Assumption 2 in [26] can be invoked to ensure that distortion and perceptual measures are not overly sensitive to discretizations.

### 3.1. Realism Constraints

We summarize a spectrum of perceptual constraints **(P)** in Table 1.

#### 3.1.1. Weak and Strong Realism Constraints

The notion of weak realism is inspired by machine learning practices, such as GAN-based image reconstruction schemes [4]. In information theory, weak realism has been studied within the framework of empirical coordination [41,42] using the empirical distribution (type). In contrast, strong realism compares joint distributions over blocks of symbols, thereby imposing stricter constraints. Matsumoto [44,45] investigated the RDP trade-off for general information sources using fixed-length and variable-length coding, suggesting the need for channel resolvability codes. Single-letter expressions can be derived under the assumption of i.i.d. output [31,43].

#### 3.1.2. Perfect and Imperfect Realism Constraints

The concept of perfect realism [25,29] is analogous to the lossless scenario in rate–distortion problems, requiring that the output distribution closely matches the source distribution in terms of a perceptual measure. This aligns closely with lossy coding under distribution constraints [31], where the output sequence must adhere to a specified i.i.d. distribution while also satisfying distortion constraints. The near-perfect realism constraint requires that the reconstruction distribution be asymptotically arbitrarily close to the source distribution. Wagner [25] demonstrated that achievability with perfect realism is equivalent to achievability with near-perfect realism when the distortion–perception pair is uniformly integrable. Specifically, deterministic coding is sufficient when D>0 and P>0 [26].

### 3.2. Information-Theoretic System Model

**Figure 2:**
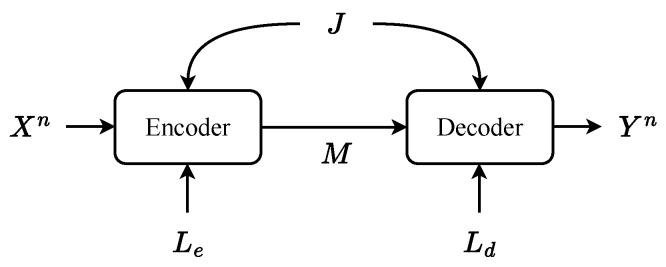
A general system model including common randomness *J*, encoder private randomness $L_{e}$, and decoder randomness $L_{d}$ as in [27].

As an example, we consider the general system model introduced in [27], which incorporates both common randomness and private randomness (see Figure 2). The encoder and the decoder have access to a shared source of randomness *J* uniformly distributed over $\left[2^{nR_{c}}\right]$. The encoder and decoder also have private randomness sources $L_{e}∼P_{L_{e}}$ and $L_{d}$ uniformly distributed over $\left[2^{nR_{d}}\right]$, respectively. The encoder observes the source $X^{n},J,L_{e}$ and selects a message $M\in [2^{nR}]$. The decoder observes the message, has access to *J* and $L_{d}$, and attempts to reconstruct the input as $Y^{n}$. Hamdi et al. [27] showed that the private randomness of the encoder is not useful under the constraint of near-perfect realism. Other variants of the system model include models with (common) side information [43,46].

**Definition 3.**  *An $\left(n,2^{nR},2^{nR_{c}},2^{nR_{d}}\right)$ fixed-length code consists of an encoding function*

$$f_{n}:X^{n}\times \left[2^{nR_{c}}\right]\times L_{d}\mapsto \left[2^{nR}\right]\;(\mathrm{possibly}\;\mathrm{stochastic})$$

*and a decoding function*

$$g_{n}:\left[2^{nR}\right]\times \left[2^{nR_{c}}\right]\times \left[2^{nR_{d}}\right]\mapsto X^{n}\;(\mathrm{possibly}\;\mathrm{stochastic}).$$

The system presented here is designed for fixed-length codes. For variable-length codes, the uniform distributions of *J* and $L_{d}$ are replaced with the following constraints:

$$R\geq \frac{1}{n}H\left(M\right),\;R_{c}\geq \frac{1}{n}H\left(J\right),\;R_{d}\geq \frac{1}{n}H\left(L_{d}\right).$$

In stochastic coding, it is assumed that various degrees of common randomness between the encoder and decoder, as well as private randomness, are available [27]. When common or private randomness is not available, the corresponding random variables $J,L_{e}$, or $L_{d}$ are set to constant, and their respective rates become zero. Specifically, when *J* and $L_{e}$ are constant, the encoding function $f_{n}$ becomes deterministic with respect to $X^{n}$. Similarly, when *J* and $L_{d}$ are constant, the decoding function $g_{n}$ becomes deterministic with respect to $X^{n}$.

### 3.3. Coding Theorems

Within the RDP framework, two scenarios, the one-shot and the asymptotic, have been extensively studied by Theis and Wagner [47] and Chen et al. [26]. The proofs for the one-shot scenario mainly rely on the strong functional representation lemma [48], whereas the proofs for the asymptotic scenario predominantly utilize techniques in channel synthesis [39,41]. Coding theorems in information theory typically involve both achievability and converse results.

**Definition 4**  (Achievablility). *The tuple $\left(R,R_{c},R_{d},D,P\right)\in R_{\geq 0}\times {\bar{R}}_{\geq 0}^{4}$ is achievable if, for any ϵ>0, there exists a sequence of $\left(n,2^{n(R+\epsilon )},2^{n(R_{c}+\epsilon )},2^{n(R_{d}+\epsilon )}\right)$ codes $\left(f_{n},g_{n}\right)$ such that the distortion constraint*

(**D**) $$E_{P}\left[\Delta \left(X^{n},Y^{n}\right)\right]\leq D+\epsilon ,$$

*and the perceptual constraint(s) **(P)** are both satisfied, where $Y^{n}=g_{n}\left(f_{n}\left(X^{n},Z^{n},K\right),Z^{n},K\right)$.*

**Definition 5.**  *The rate region $R_{D,P}$ is the closure of the set of achievable rates, i.e.,*

$$R_{D,P}=\mathrm{cl}\left(\{\left(R,R_{c},R_{d}\right)\in R_{\geq 0}\times {\bar{R}}_{\geq 0}^{2}:\left(R,R_{c},R_{d},D,P\right)\;\mathrm{is}\;\mathrm{achievable}\}\right).$$

Given a rate region $R_{D,P}$ induced by a coding scheme, the converse theorem asserts that any rate tuples $\left(R,R_{c},R_{d}\right)$ outside $R_{D,P}$ are unachievable. Proofs of the converse often leverage the time-sharing technique (e.g., see [25,31,43]), which relies on the assumption that the output sequence is i.i.d. However, the single-letter characterization can also be derived under tensorizable and decomposable conditions [26], which implicitly imply the optimality of an i.i.d. output sequence.

### 3.4. The Rate–Distortion–Perception Frontier

The rate–distortion function in the traditional (additive) distortion setting has been successful in characterizing the minimum rate required to describe a source within a specified distortion. For a (not necessarily finite) set of distortion measures ${\left\{\Delta_{\lambda }\left(\cdot ,\cdot \right)\right\}}_{\lambda \in \Lambda }$, it is shown in [49] (Exercise 7.14, Page 117) that the rate–distortion function is

(17) $$R\left(\left\{D_{\lambda }\right\}\right)=\underset{P_{\hat{X}|X}}{\inf}I\left(X;\hat{X}\right)\;s.t.\;E\left[\Delta_{\lambda }\left(X,\hat{X}\right)\right]\leq D_{\lambda }\;\forall \lambda \in \Lambda ,$$

assuming that $\inf_{D_{\lambda }\neq 0}D_{\lambda }>0$.

The information rate–distortion–perception (I-RDP) function was first proposed in analogy to the traditional RD function within the machine learning community.

**Definition 6**  (I-RDP). *Given a random variable X that represents the source distribution, the information rate–distortion–perception function [4] is defined as*

(18) $$R^{\left(I\right)}\left(D,P\right):=\underset{P_{\hat{X}|X}}{\inf}I\left(X;\hat{X}\right)\;s.t.\;E\left[\Delta \left(X,\hat{X}\right)\right]\leq D,\;D\left(P_{X},P_{\hat{X}}\right)\leq P.$$

When the perceptual constraint **(P)** is relaxed, i.e., when P=∞, the RDP function reduces to the classic RD function R(D,∞)=R(D). Efficient computation of the I-RDP function has been explored in [50,51,52].

Based on the I-RDP function, ref. [8] demonstrated that the cost of achieving perfect realism is a doubling of the lowest achievable MSE distortion, i.e.,

$$R^{\left(I\right)}\left(D,0\right)\leq R^{\left(I\right)}\left(\frac{1}{2}D,\infty \right).$$

Specifically, [53] considered the MSE distortion measure and the 2-Wasserstein distance (see Figure 1b), showing that the distortion–perception function using these measures is quadratic. In [47], the authors first established that the I-RDP function provides a lower bound on the one-shot achievable rate using the Poisson functional representation lemma [48]. Let $R^{*}\left(D,P\right)$ denote the one-shot RDP function. The study in [9] subsequently established the relationship between the information function and the operational functions using the same strategy, resulting in the following upper and lower bounds:

$$R^{\left(I\right)}\left(D,P\right)\leq R^{*}\left(D,P\right)\leq R^{\left(I\right)}\left(D,P\right)+\log\left(1+R^{\left(I\right)}\left(D,P\right)\right)+5.$$

In the asymptotic setting, the rate region of the general system illustrated in Figure 2 is given by $R_{D,0}$ as presented in [27]:

$$\begin{array}{cc} R_{D,0}=\{\left(R,R_{c},R_{d},D\right):\;\exists \;P_{XVY} & \in A\;s.t.\\ R & \geq I_{P}\left(X;V\right)\\ R+R_{c} & \geq I_{P}\left(Y;V\right)\\ R_{d} & \geq H_{P}\left(Y|V\right)\\ D & \geq E_{P}\left[\delta \left(X,Y\right)\right]\},\\ \mathrm{where}\;A:=\{P_{XVY}:\;X & ∼P_{X},\;Y∼\;P_{X}\\ X & ⊥Y\;|\;V\\ \left|V\right| & \leq {\left|X\right|}^{2}+1\}. \end{array}$$

The RDP function can be derived from the achievability and converse of the coding theorems.

**Corollary 1.**  *When common randomness and decoder randomness are unlimited, the RDP function induced by Equation (20) is given by the following:*

(19) $$R\left(D,P\right)=\underset{P_{Y|X}}{\inf}I\left(X;Y\right)\;s.t.\;E\left[\delta \left(X,Y\right)\right]\leq D,\;Y∼P_{X}.$$

This result is obtained by setting $R_{c}=R_{d}=\infty$. Consequently, the rate region becomes

(20) $$\begin{array}{cc} R_{D,0}^{\infty ,\infty } & =\{\left(R,\infty ,\infty ,D\right):\;\exists \;P_{XVY}\in A\;s.t.\;R\geq I_{P}\left(X;V\right),D\geq E_{P}\left[\delta \left(X,Y\right)\right]\}\\ \; & =\{\left(R,\infty ,\infty ,D\right):X∼P_{X},\;Y∼P_{X},R\geq I_{P}\left(X;Y\right),D\geq E_{P}\left[\delta \left(X,Y\right)\right]\}, \end{array}$$

where the equality in Equation (20) follows from the data processing inequality $I_{P}\left(X;V\right)\geq I_{P}\left(X;Y\right)$, given that X⊥Y|V in A. Therefore, the rate region $R_{D,0}^{\infty ,\infty }$ is a subset of the RD region $R_{D}$, i.e., $R_{D,0}^{\infty ,\infty }\subseteq R_{D}$.

In light of these results, we draw the following conclusions regarding the introduction of the realism constraint:

- Common randomness and private randomness are helpful in achieving (near) perfect realism.
- The introduction of the perceptual constraint results in a higher rate requirement, even when common randomness and decoder randomness are unlimited.

As illustrated in Figure 1a, where a Gaussian source is considered, the perceptual constraint modestly increases the bitrate required in theory, suggesting an increase in traffic in communication networks. The red arrows indicate the additional rates needed under conditions of no common randomness and unlimited common randomness, respectively. In practice, decoder randomness can be implemented using a random number generator, while common randomness can be achieved through a shared random seed in end-to-end training, where neural network models are deployed at both the encoder and decoder.

The information-theoretic results presented reveal that incorporating perceptual constraints necessitates a fundamental rethinking of RD analysis. Key findings include the establishment of the RDP function as a lower bound on achievable rates, the demonstration that common and private randomness can improve performance under perceptual constraints, and the characterization of how these constraints affect system design in practical communication scenarios.

## 4. Generative Modeling as a Distribution Approximation Process

The theoretical framework of RDP provides valuable insights into how generative models can be designed to balance fidelity and perceptual quality. In practice, these models must navigate complex trade-offs between maintaining statistical properties of the source data and ensuring efficient representation and transmission.

The growing demand for machine-generated content has spurred the development of generative models across various domains, including language, image, and video generation. Given that these models aim to produce content that appears realistic to humans, evaluation often relies on perceptual quality metrics, which have been integrated into several benchmarks. For instance, the PyIQA package [54] provides a convenient means of computing distortion and perceptual quality measures for image compression. This section underscores the significance of high perceptual fidelity in downstream machine learning tasks, particularly in generative modeling. By examining architectures such as VAEs, GANs, transformers, and diffusion models, along with their objectives, we demonstrate that generative modeling can be interpreted as a distribution approximation process, where the models are trained to match the distribution of the training data.

### 4.1. Objectives, Tasks, and Architectures

The objective of generative modeling is to train a neural network $P_{\theta }$ capable of generating new samples y∈X from random seeds (and conditional samples) that closely resemble those in the training data S. From a frequentist viewpoint, the observations x∈S are drawn i.i.d. from an unknown true distribution: $S={\left\{x_{i}\right\}}_{i=1}^{N}∼P_{X}.$ The goal is to learn a generator $g_{\theta }$ that can produce samples approximating $P_{X}$ by passing a random variable *U* with a fixed distribution, typically N(0,1), through the network. Since these models are trained to minimize specific perceptual objectives, the random seed *U* can be regarded as private randomness that enhances the perceptual quality of the reconstruction, conditioned on given messages. The decoding process in generative models can be viewed as a sampling procedure. For example, in the context of LLMs, beam search [55] is a common maximum a posteriori (MAP) decoding algorithm. Other sampling algorithms, such as temperature sampling [56], top-*k* sampling [57], nucleus sampling [58], and typical sampling [59], have been developed to generate content faithfully according to the learned distribution.

A variety of generative model architectures have been proposed, with the most prominent ones including variational auto-encoders (VAEs) [60], generative adversarial networks (GANs) [61], diffusion models [62,63], and transformers [64]. These models have achieved notable success in generating images, videos, and language content. They are trained by minimizing specific loss functions that quantify the similarity between the model distribution $P_{\theta }$ and the data distribution $P_{D}$. For instance, VAEs and diffusion models minimize the negative evidence lower bound (ELBO), which effectively reduces the KL divergence, while GANs optimize objectives ranging from the Jensen–Shannon divergence [61] to the Wasserstein distance [65]. At the core of generative modeling are the divergence measures $D(P_{\theta },P_{D})$ between the model and data distributions. Building on these foundations, neural networks for specific tasks, such as lossy compression, can be trained to minimize a combined distortion and perception loss with a weighting factor λ:

(21) $$L=\Delta \left(X,\hat{X}\right)+\lambda D\left(P_{X},P_{\hat{X}}\right).$$

Variants of this loss function have been applied in numerous settings, including those described in [5,6,7,8,9,10,11], to achieve high perceptual quality. These applications underscore the perceptual–distortion trade-off inherent in the training of generative models in practice. As the realism depends heavily on the statistical properties of the training datasets, different dataset statistics may affect the RDP trade-off of generative models.

#### 4.1.1. Variational Auto-Encoder

VAEs can effectively learn the distribution of data P(x) and generate samples x∼P(X) using latent variables *Z* from a prior distribution P(Z). The marginal likelihood for inference requires the integration of the latent variable P(x)=∫P(x,z)dz, which is often intractable. Therefore, an inference model $Q_{\phi }\left(z|x\right)$ and generative decoder $P_{\theta }\left(x|z\right)$ are trained end-to-end by maximizing the evidence lower bound (ELBO):

(22) $$\begin{array}{cc} \log P(x) & =\log\int Q\left(z|x\right)\frac{P(x,z)}{Q(z|x)}dz\geq \int Q\left(z|x\right)\log\left(\frac{P(x|z)P(z)}{Q(z|x)}\right)\\ \; & =E_{Q_{\phi }\left(z|x\right)}\left[\log P_{\theta }\left(x|z\right)\right]-D_{\mathrm{KL}}\left(Q_{\phi }\left(z|x\right)∥P_{\theta }\left(z\right)\right)=:L_{\mathrm{ELBO}}\left(x;\theta ,\phi \right) \end{array}$$

for continuous *z* via stochastic gradient descent. Semi-supervised VAEs [66] are capable of incorporating discrete “semantic labels” as additional variables.

#### 4.1.2. Generative Adversarial Network

The objective of a GAN is to minimize the divergence between the real data distribution $P_{X}$ and the generative model distribution $P_{G}$. For example, the vanilla GAN [67] employs the Jensen–Shannon divergence

$$D_{\mathrm{JS}}\left(P_{X},P_{Y}\right)=\frac{1}{2}D_{\mathrm{KL}}\left(P_{X}∥\frac{P_{X}+P_{Y}}{2}\right)+\frac{1}{2}D_{\mathrm{KL}}\left(P_{Y}∥\frac{P_{X}+P_{Y}}{2}\right).$$

The *f*-GAN [68] has been proposed to minimize a variational objective for different *f*-divergences:

$$d_{f}\left(P_{X}∥P_{Y}\right)=\sum_{T\in \tau }\left(E_{X}\left[T\left(X\right)\right]-E_{Y}\left[f^{*}\left(T\left(Y\right)\right)\right]\right)$$

where $f^{*}$ is the conjugate function of *f*. The Wasserstein GAN (WGAN) [65,69] minimizes the 1-Wasserstein distance. A combination of JS divergence and Wasserstein distance is proposed as the training objective in [70].

#### 4.1.3. Transformer

Transformer networks, introduced by [64], consist of a stack of homogeneous layers, including multi-head attention and feed-forward layers. The pre-training process can be interpreted as learning the energy of the data distribution using associative memory [19]. The transformer architecture has been extensively applied to language models, where the alphabet X comprises tokens that can be understood as codewords. Additionally, a distinguished symbol EOS∉X is introduced to signify the end of the sequence. Let $\bar{X}:=X\cup \left\{\mathrm{EOS}\right\}$, a language model, be parametrized by θ∈Θ, which is a (discrete) distribution $p_{\theta }$ over $X^{*}$, such that

(23) $$p_{\theta }\left(x\right)=\hat{p}\left(\mathrm{EOS}|x\right)\prod_{i=1}^{T}\hat{p}\left(x_{i}|x_{<i}\right)$$

for $x\in X^{*}$ and T=|x|, where the conditional distribution $\hat{p}\left(x|x\right)$ is a sequence model for x∈X and $x\in X^{*}$. During decoding, the next token is sampled according to a sampler $q(p_{\theta })$ to generate sequences at the decoder that approximate the distribution of the language model.

The autoregressive modeling of images using convolutional architectures has achieved success for low-resolution images. However, transformers consistently outperform their convolutional counterparts for high-resolution images [71]. Specifically, ref. [72] utilizes transformers to simplify neural video compression.

#### 4.1.4. Diffusion Model

In diffusion models [62,63], particularly score-based diffusion models [73,74], refinement information is obtained by incrementally adding white noise to the signal, thereby transforming the source distribution into a Gaussian shape after a Markov chain of diffusion steps. Compared to other image generation methods, such as generative GANs, diffusion-based image generation exhibits superior image sample quality [75].

The forward diffusion process encodes the refinement information using the following Gaussian transition kernel:

(24) $$\begin{array}{c} p_{t}\left(x_{t}|x_{t-1}\right)=N\left(x_{t};\sqrt{1-\beta_{t}}x_{t-1},\beta_{t}I\right). \end{array}$$

A learned diffusion process starts at a trivial known distribution, typically N(0,I). Furthermore, $X_{t}$ can be sampled directly according to the cumulative kernel [63], such that

(25) $$\begin{array}{c} X_{t}=\sqrt{{\bar{\alpha }}_{t}}X_{0}+\sqrt{1-{\bar{\alpha }}_{t}}\epsilon , \end{array}$$

where ${\bar{\alpha }}_{t}=\prod_{s=1}^{t}\left(1-\beta_{s}\right)$ and ϵ∼N(0,I). Therefore, the generated samples $X_{t}$ approximately follow the same distribution as the data distribution.

While diffusion models can be interpreted as a special case of VAEs with a particular choice of inference model and generative decoder, the training objective is the ELBO loss with simple data augmentation [76].

### 4.2. Practical Distortion and Perceptual Quality Measures

In practical applications such as image quality assessment, two types of metrics focus on pixel quality and statistical properties, respectively. Distortion measures, such as mean squared error (MSE), structural similarity index (SSIM), its multiscale variant (MS-SSIM) [77], and peak signal-to-noise ratio (PSNR), require a ground-truth image for comparative analysis (see Figure 1c). Traditional video codecs are designed to efficiently remove spatial–temporal redundancies. Current coding standards, including H.264/AVC [78], H.265/HEVC [79], and H.266/VVC [80], achieve efficient compression through rate–distortion optimization, where the rate is represented by bits-per-pixel (BPP) and distortion is measured by PSNR.

In contrast, perceptual quality measures focus on the statistical properties of images. For example, the Fréchet Inception distance (FID) [81] uses the Inception Network to assess image quality without a reference image by comparing the distributions of generated and real images used to train the generator. The learned perceptual image patch similarity (LPIPS) [82] computes the distance between features in the latent space of a trained neural network and has been shown to effectively capture the underlying semantics of the data. In [83], an unsupervised perceptual information metric (PIM) is proposed using information-theoretic objectives. It is common practice, especially in the domain of LLMs, to employ a powerful model as a judge of semantic similarities. This judge can be conceptualized as a function, and the optimal rate of lossless functional compression can be determined using graph entropy [84,85,86].

Learning-based compression methods not only consider pixel-wise signal quality metrics such as PSNR but also emphasize perceptual quality. These methods are increasingly outperforming standard codecs in many cases. Inspired by the information-theoretic setting [31], ref. [3] employs a GAN-based model to optimize the RD trade-off under the constraint that reconstructed samples follow the training data distribution. Source coding based on diffusion models is also gaining prominence [87,88]. In [89], a posterior sampling-based compression (PSC) method using diffusion is proposed, with two configurations: PSC-Perception and PSC-Distortion, focusing on high perceptual quality and low-distortion reconstructions, respectively. With the increasing demand for processing longer contexts, significant research has also been devoted to the RD trade-off in text compression using large language models (LLMs) [90,91,92].

### 4.3. Experimental Validation in Image Compression

To demonstrate the practical implications of our theoretical findings, we conducted experiments on image compression using the RDP framework. We implemented several learning-based compression methods that incorporate perceptual measures and compared them against traditional compression codecs.

We evaluated the performance of five compression methods: BPG (baseline), VAE-based compression [93], attention-based compression [15], GAN-based compression [7], and diffusion-based compression [88] at a low bpp regime (R≈0.2 bpp). We used the CLIC2020 dataset [94], which contains 428 high-quality color images, for our experiments. The performance metrics include PSNR, MS-SSIM, and the perceptual-oriented LPIPS.

The results are summarized in Table 2. The GAN-based method shows the most significant improvement in perceptual quality metrics while maintaining competitive distortion metrics. The attention-based method provided better PSNR than traditional methods but showed less improvement in perceptual metrics.

## 5. AI-Empowered Communication with Perceptual Constraint

The advances in generative modeling discussed thus far have significant implications for communication systems, particularly in scenarios where high perceptual quality is essential for downstream tasks. These models can be deployed at various points in the communication pipeline to enhance both compression efficiency and reconstruction quality.

Recent years have witnessed substantial progress in applying machine learning algorithms, particularly generative modeling, to communication networks, driven by their capacity to enhance perceptual quality. Downstream tasks, ranging from image reconstruction [13] to steganography [51], are highly sensitive to distribution and can be executed at the wireless edge using neural networks. Learning-based methods can more easily account for and optimize perceptual qualities as the training of these neural network models inherently addresses the distribution of the output. Thus, perceptual requirements are both natural and essential for AI-empowered communication systems.

### 5.1. Source–Channel Coding for Perceptual Oriented Communications

**Figure 3:**
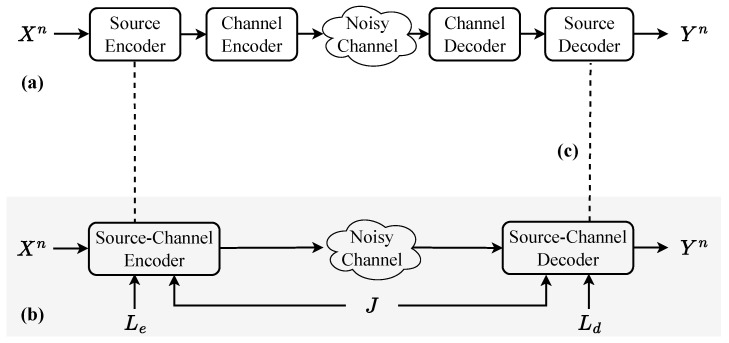
(**a**) Separate source and channel coding (SSCC) in current communication system. (**b**) Joint source–channel coding (JSCC) scheme as in [13]. (**c**) The dashed lines link SSCC and JSCC to form a hybrid communication scheme, as in [105].

Current digital communication systems are designed based on Shannon’s source–channel separation theorem [95], which posits that there is no loss of optimality in applying separate source coding followed by channel coding in the asymptotic infinite block length regime, provided that the source and channel statistics are ergodic and the distortion measure is additive. As shown in Figure 3a, the source-encoded data are transmitted through the wireless channel after channel coding and modulation. The receiver reverses these procedures by first demodulating and decoding the channel code, followed by decompression to reconstruct the original data.

While the optimality of separation holds for distortion–perception measures under assumptions of perceptual measures, including sub-decomposability, it has been demonstrated that separation is no longer optimal when common randomness between the encoder and decoder is unavailable [32]. Moreover, in practice, idealized assumptions, such as memorylessness, are rarely met [96], rendering separation-based digital communication systems suboptimal [97]. This motivates the adoption of joint source–channel coding (JSCC) to enhance end-to-end performance in practical communication systems (see Figure 3b). Recently, in the context of semantic communications [98], deep-learning-based JSCC methods, such as DeepJSCC [13], have achieved remarkable results by learning mappings directly from training data (both source and channel) [13,99,100,101,102,103,104]. Furthermore, a hybrid scheme that combines SSCC and JSCC has been proposed in [105] (see Figure 3c), demonstrating significant potential in mediating between the two systems.

### 5.2. AI-Empowered Semantic Communication

Driven by advances in machine learning, there has been a significant increase in interest in semantic communication systems [98,106,107], with intelligent network architectures being progressively integrated into existing communication standards [108,109,110,111,112,113]. Broadly, the objective of communication is to transmit meaningful information. In the context of generative models, the term “meaningful” is contingent upon the semantic meaning of the information, which represents a fundamentally different global property compared to traditional average distortion measures, such as Hamming distance and MSE. The notions of semantic and pragmatic communication problems are addressed by drawing parallels to linguistics [107]. By integrating machine learning algorithms, particularly generative modeling, into the widely deployed digital communication infrastructure, semantic communication aims to deliver content with higher perceptual quality more robustly over wireless channels, which are frequently influenced by environmental factors such as weather conditions, buildings, and device activities.

#### 5.2.1. Theoretical Foundation and Practical Motivation

In the RDP framework, common and private randomness have been shown to play crucial roles in improving perceptual fidelity while maintaining efficient communication. Theoretically, common randomness (shared between the transmitter and receiver) and private randomness (available only to one node) provide additional degrees of freedom that can be leveraged to optimize the trade-off between rate, distortion, and perception. In practical systems, this can be implemented through several methods.

##### Shared Random Seeds in End-to-End Training

In learning-based communication systems, common randomness can be implemented by sharing random seeds between the transmitter and receiver during the training phase. For example, in systems using VAEs or GANs, the encoder and decoder can be trained with a shared random seed that initializes their respective random number generators. This ensures that both ends of the communication system can generate correlated random variations, which can be used to improve perceptual quality.

##### Pilot Signals and Synchronization

In wireless communication systems, common randomness can be established through pilot signals transmitted from the base station to user equipment. These pilot signals can be used to synchronize random number generators at both ends of the communication link. For instance, in massive MIMO systems, the base station can transmit reference signals that user equipment uses to align its random seed with the base station’s generator.

##### Implementation of Private Randomness

Both transmitters and receivers can incorporate local random number generators that do not require coordination with the other end of the communication system. These generators can be based on physical phenomena (such as thermal noise) or algorithmic approaches (such as pseudorandom number generators with locally maintained seeds).

**Figure 4:**
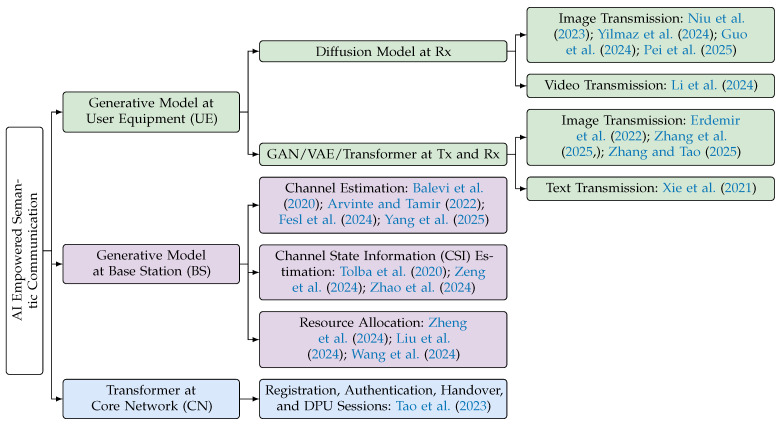
Generative models deployed on user equipment (UE), base stations (BSs), and core network (CN) in AI-empowered communication [105,114,115,116,117,118,119,120,121,122,123,124,125,126,127,128,129,130,131,132,133].

In the following, we identify three use cases where generative models can be deployed on user equipment (UE), base stations (BSs), and the core network (CN), as illustrated in Figure 4.

#### 5.2.2. Generative Modeling in User Equipment

Due to the increasing hardware computing capability, UE such as smartphones is increasingly capable of performing intelligent tasks involving generative models. In wireless communications, UE encompasses a wide range of devices, including smartphones, tablets, specialized Internet of Things (IoT) devices, and industrial sensors, all of which are capable of transmitting and receiving reliable and efficient communication services. UE features software-defined encoders and decoders for audio, video, and other data formats, as well as support for wireless communication protocols such as LTE and 5G NR. More recently, LLMs have been deployed on edge devices, such as smartphones. Applications like prompt compression [92] enable extended model inference (EMI) and native model inference (NMI), which apply prompt compression and inference on the same devices and facilitate interactions between edge devices and cloud servers, respectively.

Recent research indicates that the joint source–channel coding (JSCC) scheme, combined with a generative model for reconstruction, can achieve substantial reductions in bandwidth while significantly enhancing the perceptual quality of the reconstruction. Utilizing VAEs, ref. [12] reimagines communication systems as a joint optimization of the transmitter (Tx) and receiver (Rx) in an end-to-end manner for reconstruction tasks. The source node (Tx) encodes the data using neural networks that create compact representations while ensuring reliability. For example, a pre-trained generative model based on GANs is employed in [126], and the destination node (Rx) utilizes a generative model to reconstruct the data. In [131], a diffusion model is deployed at the receiver to achieve high-perceptual-quality wireless image transmission. In [134], common modulation schemes are incorporated into the encoded representation as well. In [100], a JSCC video transmission scheme is proposed using deep neural networks. Additionally, Rx may provide feedback to the source node, enabling dynamic adjustments to enhance communication quality [135].

#### 5.2.3. Generative Modeling in Base Station

As part of the radio Access Network (RAN), the BS possesses powerful Tx and Rx capabilities to communicate simultaneously with multiple UE. Generative models have been applied to the BS for channel estimation, channel state information (CSI) acquisition, and resource allocation.

The BS features encoders and decoders to process both uplink (from UE to the BS) and downlink (from the BS to UE) data streams. Modern cellular networks typically operate in full-duplex mode [136], enabling simultaneous bidirectional communication between UE and the BS for concurrent transmission and reception. Antenna arrays in the BS are used to send and receive radio signals, employing sophisticated beamforming techniques. The BS can estimate the current state of the channel, thereby optimizing downlink data transmission. In frequency division duplexing systems, the CSI is fed back to the BS for beamforming. In [118], a GAN-based CSI feedback approach is proposed, where the encoder learns a compressed codeword of the CSI and passes it to the generator for reconstruction in the BS. In [119], a CSI-GPT framework is proposed to efficiently acquire downlink CSI information in massive MIMO systems. In [137], the generalization ability of multi-modal large models across diverse base station environments is explored for various CSI-based tasks. Intelligent algorithms [115,116,117] can be applied to resource allocation and scheduling to dynamically allocate frequency, time, and power resources, thereby meeting real-time user needs and maximizing overall system performance.

#### 5.2.4. Generative Modeling in Core Network

The core network may include multiple types of encoders and decoders to handle data from different protocol stack layers, such as voice encoding and video encoding, to meet diverse service requirements and quality of service (QoS) standards. In [114], two models, 5GCSeq2Seq and 5GC-former, are proposed based on the vanilla Seq2Seq model and the transformer, respectively, to accurately replicate the principal functionalities of the 5G core network control plane, including registration, authentication, handover, and DPU sessions.

#### 5.2.5. Basic Unit for AI-Empowered Communications

In contemporary communication networks, formatted blocks of data are transmitted across various layers of the protocol stack. For instance, in the physical layer of 5G networks, binary digits (bits) are modulated and transmitted over the air interface. In the transport layer, messages from applications are encapsulated into segments such as TCP segments, which include headers, source and destination port numbers, and checksums. This segmentation focuses on the accurate transmission of application messages, with the goal of minimizing the average distortion measure, which is additive in terms of segmental distortion.

In DeepJSCC-like systems, analog signals are employed for end-to-end uncoded transmission [13,135]. Ref. [134] further integrates uncoded signals into a digital modulation system, which is then transmitted and received by the decoder. In the context of low-latency transmission, where information freshness is crucial, short packets serve as the basic unit [138], and the age–distortion trade-off has been studied [139]. Ref. [105] combines analog signals for JSCC with conventional digital signals to form a hybrid communication scheme.

In many generative modeling architectures, tokens function as the fundamental unit. Ref. [140] introduces a token-domain multiple-access scheme, which allows multiple devices to transmit over the same multiple-access channel in a non-orthogonal manner. In the realm of source coding, ref. [141] utilizes 1D tokenization to project 2D images into variable-length, ordered 1D token sequences. This method describes images in a coarse-to-fine manner, thereby achieving a trade-off between the number of tokens (rate) and perceptual quality. The SoTA tokenizer FlowMo is a transformer-based diffusion autoencoder that combines mode matching pre-training and mode seeking post-training [142]. In [143], a video tokenizer is developed for both videos and images using a common token vocabulary. These systems are trained end-to-end using losses that incorporate perceptual quality. Furthermore, technologies such as chunking and the semantic compression of token chunks have been developed in [90], leveraging the parallel computational power of GPUs. In this approach, chunks of tokens serve as the fundamental unit. Given the diverse representation of information, the integration of signals such as tokens and architectures such as tokenizers into intelligent communication systems is necessitated through the lens of the RDP framework.

The integration of generative models into communication systems demonstrates substantial potential for improving perceptual quality while maintaining efficient transmission. Joint source–channel coding approaches like DeepJSCC have shown particular promise by directly optimizing for end-to-end reconstruction quality rather than treating source and channel coding as separate problems. However, practical implementation faces challenges related to computational complexity, training data requirements, and the need for robustness against varying channel conditions.

## 6. Conclusions and Future Direction

### 6.1. Conclusions

This paper has explored the critical role of perceptual constraints in information theory and their impact on practical generative modeling scenarios. We have demonstrated that incorporating perceptual constraints into the RD framework, resulting in the RDP framework, offers a novel and comprehensive perspective for evaluating the performance of AI-empowered systems in compression and communication. We have discussed the implications of the perceptual constraint on the design and deployment of generative models, which is particularly relevant in intelligent communication systems, where downstream tasks demand high perceptual fidelity, such as interactive AI agents and visually pleasing content. By highlighting the importance of perceptual constraints and demonstrating their impact on system design and performance, we project an increase in expected traffic in intelligent communication networks with consideration of perceptual quality. While our theoretical analysis suggests that incorporating perceptual constraints generally requires additional rate, we acknowledge that intelligent compression techniques can significantly mitigate this traffic growth.

### 6.2. Future Direction

#### 6.2.1. Information-Theoretic Directions

In [31,43], it is assumed that the output follows the i.i.d. distribution for i.i.d. sources. When imposing the perfect realism constraint, the i.i.d. condition is naturally imposed on the output. However, it is unclear whether this assumption is valid or redundant. Therefore, we pose the following conjecture for further investigation.

**Conjecture 1.**  *Consider the system model in Figure 2 and the $\left(n,2^{nR},2^{nR_{c}},2^{nR_{d}}\right)$ fixed-length code with encoder and decoder $\left(f_{n},g_{n}\right)$. For i.i.d. source ${\left\{X_{i}\right\}}_{i=1}^{\infty }$, suppose that the distortion and perceptual constraints are*

(**D**) $$\begin{array}{cc} E_{P}\left[\Delta \left(X^{n},Y^{n}\right)\right] & \leq D, \end{array}$$

(**P**) $$\begin{array}{cc} D(P_{X^{n}},P_{Y^{n}}) & \leq P, \end{array}$$

*where $Y^{n}=g_{n}\left(f_{n}\left(X^{n},Z^{n},K\right),Z^{n},K\right)$. Then, the capacity achieving $Y^{n}$ is also i.i.d. when n→∞ for perception measures D(·,·) that belong to the f-divergence family.*

Analogously to the RD function with the multiple-distortion constraint, we pose the following conjecture.

**Conjecture 2.**  *For a (not necessarily finite) set of distortion measures ${\left\{\Delta_{\lambda }\left(\cdot ,\cdot \right)\right\}}_{\lambda \in \Lambda }$ and a (not necessarily finite) set of f-divergences ${\left\{D_{f_{\gamma }}\left(\cdot ,\cdot \right)\right\}}_{\gamma \in \Gamma }$, suppose that the source ${\left\{X_{i}\right\}}_{i=1}^{\infty }$ and reconstruction Y are from the same discrete alphabet X. Consider the (possibly stochastic) code $\left(f_{n},g_{n}\right)$; then, the RDP function with unlimited common randomness and unlimited private randomness is*

(26) $$\begin{array}{cc} R(\left\{D_{\lambda }\right\},\left\{P_{\gamma }\right\}) & =\underset{P_{Y|X}}{\inf}I\left(X;Y\right)\\ s.t.\; & E\left[\Delta_{\lambda }\left(X,Y\right)\right]\leq D_{\lambda },\;\forall \lambda \in \Lambda \\ \; & D_{f_{\gamma }}\left(P_{X^{n}},P_{Y^{n}}\right)\leq P_{\gamma },\;\forall \gamma \in \Gamma \end{array}$$

*where $\inf_{D_{\lambda }\neq 0}D_{\lambda }>0$ and $\inf_{P_{\gamma }\neq 0}P_{\gamma }>0$.*

When the realism constraint is defined in terms of weak realism, the conjecture can be proven using the method of types [42]. However, it remains unclear whether the conjecture holds under other realism constraints, such as strong realism, or under a combination of different constraints.

#### 6.2.2. Architectural Improvement for Intelligent Communication Systems

Future architectural designs for semantic communications have been discussed in [144,145,146,147]. The integration of perceptual fidelity into the design of intelligent communication architectures represents a paradigm shift from traditional communication systems optimized solely for distortion. The development of systems that better align with human-centric quality assessments and support emerging applications requiring high perceptual fidelity is a promising direction for future research and development, particularly as applications increasingly demand high perceptual quality in transmitted data. By bridging the gap between theoretical advances in information theory and practical deployment considerations, the RDP framework can guide intelligent communication architectures that better serve human-centric needs.

##### Interactive AI Agents

In interactive AI agent scenarios, communication systems must support real-time decision making while maintaining perceptual consistency with human expectations. RDP trade-off provides a theoretical foundation for balancing the rate requirements against both distortion and perceptual constraints in these dynamic environments. The deployment of generative models at both transmitter and receiver nodes allows for the incorporation of common and private randomness, which has been shown to improve perceptual fidelity in RDP frameworks. For interactive agents, this translates to more natural human–AI interactions where the AI can generate responses that are not only factually accurate but also perceptually coherent with the user’s expectations. Architecturally, this requires communication systems that can dynamically adjust their rate allocation based on the perceptual importance of different components of the transmitted information. For example, in multi-modal communication systems involving text, image, and video data, the RDP function can guide the allocation of bandwidth to prioritize perceptually critical features across different data types.

##### Perceptually Optimized Data Transmission

RDP frameworks enable the design of communication systems that explicitly optimize for perceptual quality rather than just fidelity to the original data. This is particularly important in applications where the end-user experience depends on how natural/realistic the reconstructed data appear rather than on strict pixel-wise accuracy. In image and video transmission, for example, RDP-informed architectures can prioritize the preservation of distributions that contribute significantly to human perception of quality, even if this requires slightly higher bitrates. Conversely, they can allocate fewer bits to components that have minimal impact on perceptual quality, achieving more efficient compression. For example, systems employing generative models like GANs can achieve high perceptual quality with manageable increases in bitrate by leveraging the models’ ability to capture the underlying data distribution.

Reinforcement learning has emerged as a potentially transformative approach for developing agent-based systems, including embodied AI, as downstream tasks for intelligent communications. Recently, the rate–reward trade-off has been introduced [148]. Moreover, the remote coordination problem has been introduced [149], driven by the necessity to develop coordinated policies among distributed agents [150]. In this context, exploring the fundamental limits of coordination under rate-constrained communication emerges as a significant area of investigation.

##### Human-Centric Quality Systems

Communication systems prioritizing human-centric quality should account for the subjective nature of human perception, which often differs from objective distortion metrics in current system design. This requires the development of communication architectures that can adapt to the specific perceptual requirements of different applications and user contexts. For example, in tele-medicine applications, the communication system might prioritize the accurate transmission of fine details in medical images that are critical for diagnosis, while, in video conferencing, the system might focus on maintaining smooth motion and natural facial expressions. From an implementation perspective, future design should consider adaptability through modular architectures where different perceptual quality modules can be activated based on the application requirements. These modules could employ specialized generative models trained on task-specific data to optimize the perceptual quality for particular use cases.

#### 6.2.3. Implementation Challenges in Deploying Generative Models for Intelligent Communications

While generative models offer significant theoretical advantages for intelligent communication systems, practical implementation presents several challenges that must be addressed for successful deployment in real-world scenarios.

##### Computational Complexity

The computational requirements for training and deploying generative models represent a significant implementation challenge. VAEs, GANs, and diffusion models each have distinct computational profiles. VAEs typically require substantial computational resources for training, particularly when dealing with high-dimensional data such as images or video. The encoding and decoding processes involve complex neural network operations that can be computationally intensive. The adversarial training process for GANs between generator and discriminator networks can lead to increased computational complexity. The need for careful balancing between the two networks adds to the computational burden during training. Diffusion models generally require the most computational resources due to their iterative denoising process. The multiple forward and backward passes during both training and inference phases result in higher computational demands compared to VAEs and GANs. The following directions are important for addressing these challenges.

- Model architecture simplification through techniques like depthwise separable convolutions and reduced latent space dimensions.
- Knowledge distillation methods where smaller models are trained to mimic the behavior of larger, more complex generative models.
- The quantization of model weights and activations to reduce precision requirements and computational intensity.

##### Latency Considerations

Real-time communication systems often have strict latency requirements that must be met for effective operation. The latency introduced by generative models can impact system performance in end-to-end latency, model inference time, and transmission delay: the total delay from the input to output must be minimized, especially in interactive applications like video conferencing or augmented reality; the time required to generate or reconstruct data at the receiver must be compatible with the system’s real-time constraints; the additional bits required for perceptual quality may increase transmission time, creating a trade-off between quality and latency. Future directions include model architecture design specifically targeting low-latency inference, such as using lightweight models.

##### Hardware Constraints

The hardware limitations of communication devices significantly impact the deployment of generative models. Many communication systems rely on edge devices with limited computational capabilities, memory, and power. Deploying generative models on these devices requires careful optimization. While specialized hardware can mitigate some computational limitations, it introduces additional design considerations and potential costs. Moreover, in wireless communication networks, energy consumption is a critical concern. Generative models should be optimized to operate within the power constraints of battery-powered devices. Future research exploring various solutions to these hardware constraints include:

- On-device model compression techniques, including pruning and quantization.
- Federated learning approaches where model training is distributed across multiple devices.
- Hybrid architectures that perform partial processing at the edge and partial processing in the cloud.

## Figures and Tables

**Table 1:** Taxonomy of realism constraints **(P)**.

Realism Constraint	Definition	References
Weak realism	$D({\hat{P}}_{X^{n}},{\hat{P}}_{Y^{n}})\leq P$	[41,42,43]
Per-symbol realism	$D\left(P_{X},P_{Y_{t}}\right)\leq P,\;\forall t\in \left[n\right]$	[26]
Average marginal realism	$\sup_{n}\frac{1}{n}\sum_{t=1}^{n}D\left(P_{X},P_{Y_{t}}\right)\leq P$	[9]
Strong realism	$D(P_{X^{n}},P_{Y^{n}})\leq P$	[43,44,45]
Perfect strong realism	$Y^{n}∼P_{X^{n}}$	[25,31,39]
Near-perfect per-symbol realism	$\lim_{n\to \infty }\max_{1\leq t\leq n}D\left(P_{X},P_{Y_{t}}\right)=0$	[26,27]
Near-perfect strong realism	$\lim_{n\to \infty }D\left(P_{X^{n}},P_{Y^{n}}\right)=0$	[25,27]

**Table 2:** Comparison of compression methods on the CLIC2020 dataset.

Method (R≈0.2 bpp)	PSNR (↑)	MS-SSIM (↑)	LPIPS (↓)
BPG	33	0.96	0.20
Attention-based [15]	**34**	**0.97**	0.14
VAE-based [93]	31	0.95	0.20
GAN-based [7]	32	**0.97**	**0.06**

## Data Availability

The original data presented in the study are openly available in CLIC2020 at [94].

## References

[1] Blau Y., Michaeli T. The perception-distortion tradeoff. Proceedings of the IEEE Conference on Computer Vision and Pattern Recognition.

[2] Dahl R., Norouzi M., Shlens J. Pixel recursive super resolution. Proceedings of the IEEE International Conference on Computer Vision.

[3] Tschannen M., Agustsson E., Lucic M. (2018). Deep generative models for distribution-preserving lossy compression. Adv. Neural Inf. Process. Syst..

[4] Blau Y., Michaeli T. Rethinking lossy compression: The rate-distortion-perception tradeoff. Proceedings of the International Conference on Machine Learning. PMLR.

[5] Ledig C., Theis L., Huszár F., Caballero J., Cunningham A., Acosta A., Aitken A., Tejani A., Totz J., Wang Z. Photo-realistic single image super-resolution using a generative adversarial network. Proceedings of the IEEE Conference on Computer Vision and Pattern Recognition.

[6] Agustsson E., Tschannen M., Mentzer F., Timofte R., Gool L.V. Generative adversarial networks for extreme learned image compression. Proceedings of the IEEE/CVF International Conference on Computer Vision.

[7] Mentzer F., Toderici G.D., Tschannen M., Agustsson E. (2020). High-fidelity generative image compression. Adv. Neural Inf. Process. Syst..

[8] Yan Z., Wen F., Ying R., Ma C., Liu P. On perceptual lossy compression: The cost of perceptual reconstruction and an optimal training framework. Proceedings of the International Conference on Machine Learning. PMLR.

[9] Zhang G., Qian J., Chen J., Khisti A. (2021). Universal rate-distortion-perception representations for lossy compression. Adv. Neural Inf. Process. Syst..

[10] Yan Z., Wen F., Liu P. Optimally Controllable Perceptual Lossy Compression. Proceedings of the International Conference on Machine Learning. PMLR.

[11] Salehkalaibar S., Phan T.B., Chen J., Yu W., Khisti A. (2023). On the choice of perception loss function for learned video compression. Adv. Neural Inf. Process. Syst..

[12] O’shea T., Hoydis J. (2017). An introduction to deep learning for the physical layer. IEEE Trans. Cogn. Commun. Netw..

[13] Bourtsoulatze E., Burth Kurka D., Gündüz D. (2019). Deep Joint Source-Channel Coding for Wireless Image Transmission. IEEE Trans. Cogn. Comms. Netw..

[14] Bégaint J., Racapé F., Feltman S., Pushparaja A. (2020). CompressAI: A PyTorch library and evaluation platform for end-to-end compression research. arXiv.

[15] Cheng Z., Sun H., Takeuchi M., Katto J. Learned image compression with discretized gaussian mixture likelihoods and attention modules. Proceedings of the IEEE/CVF conference on Computer Vision and Pattern Recognition.

[16] Gregory R.L. (1980). Perceptions as hypotheses. Philos. Trans. R. Soc. Lond. B Biol. Sci..

[17] Rao R.P., Ballard D.H. (1999). Predictive coding in the visual cortex: A functional interpretation of some extra-classical receptive-field effects. Nat. Neurosci..

[18] Jiang L.P., Rao R.P. (2022). Predictive Coding Theories of Cortical Function. https://oxfordre.com/neuroscience/display/10.1093/acrefore/9780190264086.001.0001/acrefore-9780190264086-e-328.

[19] Niu X., Bai B., Deng L., Han W. (2024). Beyond Scaling Laws: Understanding Transformer Performance with Associative Memory. arXiv.

[20] Lin B., Ye Y., Zhu B., Cui J., Ning M., Jin P., Yuan L. Video-LLaVA: Learning United Visual Representation by Alignment Before Projection. Proceedings of the 2024 Conference on Empirical Methods in Natural Language Processing.

[21] Fei W., Niu X., Xie G., Zhang Y., Bai B., Deng L., Han W. (2024). Retrieval meets reasoning: Dynamic in-context editing for long-text understanding. arXiv.

[22] Meng C., He Y., Song Y., Song J., Wu J., Zhu J.Y., Ermon S. SDEdit: Guided Image Synthesis and Editing with Stochastic Differential Equations. Proceedings of the International Conference on Learning Representations.

[23] Cover T.M. (1999). Elements of Information Theory.

[24] Theis L., Agustsson E. (2021). On the advantages of stochastic encoders. arXiv.

[25] Wagner A.B. (2022). The Rate-Distortion-Perception Tradeoff: The Role of Common Randomness. arXiv.

[26] Chen J., Yu L., Wang J., Shi W., Ge Y., Tong W. (2022). On the Rate-Distortion-Perception Function. IEEE J. Sel. Areas Inf. Theory.

[27] Hamdi Y., Wagner A.B., Gündüz D. (2024). The Rate-Distortion-Perception Trade-off: The Role of Private Randomness. arXiv.

[28] Li M., Klejsa J., Kleijn W.B. (2010). Distribution preserving quantization with dithering and transformation. IEEE Signal Process. Lett..

[29] Li M., Klejsa J., Kleijn W.B. (2011). On distribution preserving quantization. arXiv.

[30] Saldi N., Linder T., Yüksel S. (2014). Randomized quantization and source coding with constrained output distribution. IEEE Trans. Inf. Theory.

[31] Saldi N., Linder T., Yüksel S. (2015). Output constrained lossy source coding with limited common randomness. IEEE Trans. Inf. Theory.

[32] Tian C., Chen J., Narayanan K. (2025). Source-Channel Separation Theorems for Distortion Perception Coding. arXiv.

[33] Qiu Y., Wagner A.B., Ballé J., Theis L. (2024). Wasserstein distortion: Unifying fidelity and realism. Proceedings of the 2024 58th Annual Conference on Information Sciences and Systems (CISS).

[34] Marton K. (1996). Bounding *d*¯-distance by informational divergence: A method to prove measure concentration. Ann. Probab..

[35] Marton K. (1998). Measure concentration for a class of random processes. Probab. Theory Relat. Fields.

[36] Yassaee M.H., Aref M.R., Gohari A. (2014). Achievability proof via output statistics of random binning. IEEE Trans. Inf. Theory.

[37] Pinsker M.S. (1964). Information and information stability of random variables and processes. J. R. Stat. Soc. Ser. Appl. Stat..

[38] Csiszár I., Talata Z. (2006). Context tree estimation for not necessarily finite memory processes, via BIC and MDL. IEEE Trans. Inf. Theory.

[39] Cuff P. (2013). Distributed channel synthesis. IEEE Trans. Inf. Theory.

[40] Villani C. (2009). Optimal Transport: Old and New.

[41] Cuff P.W., Permuter H.H., Cover T.M. (2010). Coordination capacity. IEEE Trans. Inf. Theory.

[42] Raginsky M. (2012). Empirical processes, typical sequences, and coordinated actions in standard Borel spaces. IEEE Trans. Inf. Theory.

[43] Niu X., Gündüz D., Bai B., Han W. (2023). Conditional Rate-Distortion-Perception Trade-Off. Proceedings of the 2023 IEEE International Symposium on Information Theory (ISIT).

[44] Matsumoto R. (2018). Introducing the perception-distortion tradeoff into the rate-distortion theory of general information sources. IEICE Commun. Express.

[45] Matsumoto R. (2019). Rate-distortion-perception tradeoff of variable-length source coding for general information sources. IEICE Commun. Express.

[46] Hamdi Y., Gündüz D. (2023). The Rate-Distortion-Perception Trade-off with Side Information. Proceedings of the 2023 IEEE International Symposium on Information Theory (ISIT).

[47] Theis L., Wagner A.B. (2021). A coding theorem for the rate-distortion-perception function. arXiv.

[48] Li C.T., El Gamal A. (2018). Strong functional representation lemma and applications to coding theorems. IEEE Trans. Inf. Theory.

[49] Csiszár I., Körner J. (2011). Information Theory: Coding Theorems for Discrete Memoryless Systems.

[50] Chen C., Niu X., Ye W., Wu S., Bai B., Chen W., Lin S.J. (2023). Computation of rate-distortion-perception functions with Wasserstein barycenter. Proceedings of the 2023 IEEE International Symposium on Information Theory (ISIT).

[51] Chen C., Niu X., Ye W., Wu H., Bai B. (2024). Computation and Critical Transitions of Rate-Distortion-Perception Functions with Wasserstein Barycenter. arXiv.

[52] Serra G., Stavrou P.A., Kountouris M. (2024). Computation of the Multivariate Gaussian Rate-Distortion-Perception Function. Proceedings of the 2024 IEEE International Symposium on Information Theory (ISIT).

[53] Freirich D., Michaeli T., Meir R. (2021). A theory of the distortion-perception tradeoff in wasserstein space. Adv. Neural Inf. Process. Syst..

[54] Chen C., Mo J. (2022). IQA-PyTorch: PyTorch Toolbox for Image Quality Assessment. https://github.com/chaofengc/IQA-PyTorch.

[55] Graves A. (2012). Sequence transduction with recurrent neural networks. arXiv.

[56] Ackley D.H., Hinton G.E., Sejnowski T.J. (1985). A learning algorithm for Boltzmann machines. Cogn. Sci..

[57] Fan A., Lewis M., Dauphin Y. Hierarchical Neural Story Generation. Proceedings of the 56th Annual Meeting of the Association for Computational Linguistics (Volume 1: Long Papers).

[58] Holtzman A., Buys J., Du L., Forbes M., Choi Y. The Curious Case of Neural Text Degeneration. Proceedings of the International Conference on Learning Representations.

[59] Meister C., Pimentel T., Wiher G., Cotterell R. (2023). Locally typical sampling. Trans. Assoc. Comput. Linguist..

[60] Kingma D.P., Welling M. Auto-encoding variational Bayes. Proceedings of the 2nd International Conference on Learning Representations.

[61] Goodfellow I., Pouget-Abadie J., Mirza M., Xu B., Warde-Farley D., Ozair S., Courville A., Bengio Y. Generative adversarial nets. Proceedings of the Advances in Neural Information Processing Systems.

[62] Sohl-Dickstein J., Weiss E., Maheswaranathan N., Ganguli S. Deep Unsupervised Learning using Nonequilibrium Thermodynamics. Proceedings of the International Conference on Machine Learning (ICML).

[63] Ho J., Jain A., Abbeel P. Denoising Diffusion Probabilistic Models. Proceedings of the Advances in Neural Information Processing Systems (NeurIPS).

[64] Vaswani A., Shazeer N., Parmar N., Uszkoreit J., Jones L., Gomez A.N., Kaiser Ł., Polosukhin I. (2017). Attention is all you need. Adv. Neural Inf. Process. Syst..

[65] Arjovsky M., Chintala S., Bottou L. Wasserstein generative adversarial networks. Proceedings of the International Conference on Machine Learning. PMLR.

[66] Kingma D.P., Mohamed S., Jimenez Rezende D., Welling M. (2014). Semi-supervised learning with deep generative models. Adv. Neural Inf. Process. Syst..

[67] Goodfellow I., Pouget-Abadie J., Mirza M., Xu B., Warde-Farley D., Ozair S., Courville A., Bengio Y. (2020). Generative adversarial networks. Commun. ACM.

[68] Nowozin S., Cseke B., Tomioka R. f-GAN: Training generative neural samplers using variational divergence minimization. Proceedings of the Advances in Neural Information Processing Systems.

[69] Gulrajani I., Ahmed F., Arjovsky M., Dumoulin V., Courville A.C. Improved training of Wasserstein GANs. Proceedings of the Advances in Neural Information Processing Systems.

[70] Farnia F., Tse D. A convex duality framework for GANs. Proceedings of the Advances in Neural Information Processing Systems.

[71] Esser P., Rombach R., Ommer B. Taming transformers for high-resolution image synthesis. Proceedings of the IEEE/CVF Conference on Computer Vision and Pattern Recognition.

[72] Mentzer F., Toderici G., Minnen D., Hwang S.J., Caelles S., Lucic M., Agustsson E. VCT: A video compression transformer. Proceedings of the 36th International Conference on Neural Information Processing Systems.

[73] Song Y., Ermon S. Generative Modeling by Estimating Gradients of the Data Distribution. Proceedings of the Advances in Neural Information Processing Systems (NeurIPS).

[74] Song Y., Ermon S. Improved Techniques for Training Score-Based Generative Models. Proceedings of the Advances in Neural Information Processing Systems (NeurIPS).

[75] Dhariwal P., Nichol A. Diffusion Models Beat GANs on Image Synthesis. Proceedings of the Advances in Neural Information Processing Systems (NeurIPS).

[76] Kingma D., Gao R. (2023). Understanding diffusion objectives as the elbo with simple data augmentation. Adv. Neural Inf. Process. Syst..

[77] Wang Z., Simoncelli E.P., Bovik A.C. (2003). Multiscale structural similarity for image quality assessment. Proceedings of the Thrity-Seventh Asilomar Conference on Signals, Systems & Computers.

[78] Wiegand T., Sullivan G.J., Bjontegaard G., Luthra A. (2003). Overview of the H. 264/AVC video coding standard. IEEE Trans. Circuits Syst. Video Technol..

[79] Sullivan G.J., Ohm J.R., Han W.J., Wiegand T. (2012). Overview of the high efficiency video coding (HEVC) standard. IEEE Trans. Circuits Syst. Video Technol..

[80] Bross B., Wang Y.K., Ye Y., Liu S., Chen J., Sullivan G.J., Ohm J.R. (2021). Overview of the versatile video coding (VVC) standard and its applications. IEEE Trans. Circuits Syst. Video Technol..

[81] Heusel M., Ramsauer H., Unterthiner T., Nessler B., Hochreiter S. (2017). GANs trained by a two time-scale update rule converge to a local nash equilibrium. Adv. Neural Inf. Process. Syst..

[82] Zhang R., Isola P., Efros A.A., Shechtman E., Wang O. The unreasonable effectiveness of deep features as a perceptual metric. Proceedings of the IEEE Conference on Computer Vision and Pattern Recognition.

[83] Bhardwaj S., Fischer I., Ballé J., Chinen T. An unsupervised information-theoretic perceptual quality metric. Proceedings of the Advances in Neural Information Processing Systems.

[84] Alon N., Orlitsky A. (1996). Source coding and graph entropies. IEEE Trans. Inf. Theory.

[85] Harangi V., Niu X., Bai B. (2023). Conditional graph entropy as an alternating minimization problem. IEEE Trans. Inf. Theory.

[86] Harangi V., Niu X., Bai B. (2023). Generalizing Körner’s graph entropy to graphons. Eur. J. Comb..

[87] Theis L., Salimans T., Hoffman M.D., Mentzer F. (2022). Lossy compression with Gaussian diffusion. arXiv.

[88] Yang R., Mandt S. (2023). Lossy image compression with conditional diffusion models. Adv. Neural Inf. Process. Syst..

[89] Elata N., Michaeli T., Elad M. (2024). Zero-Shot Image Compression with Diffusion-Based Posterior Sampling. arXiv.

[90] Fei W., Niu X., Zhou P., Hou L., Bai B., Deng L., Han W. Extending Context Window of Large Language Models via Semantic Compression. Proceedings of the Findings of the Association for Computational Linguistics: ACL 2024, Association for Computational Linguistics.

[91] Arda E., Yener A. (2025). A Rate-Distortion Framework for Summarization. arXiv.

[92] Fei W., Niu X., Xie G., Liu Y., Bai B., Han W. (2025). Efficient Prompt Compression with Evaluator Heads for Long-Context Transformer Inference. arXiv.

[93] Ballé J., Minnen D., Singh S., Hwang S.J., Johnston N. Variational image compression with a scale hyperprior. Proceedings of the International Conference on Learning Representations.

[94] Toderici G., Theis L., Johnston N., Agustsson E., Mentzer F., Ballé J., Shi W., Timofte R. (2020). CLIC 2020: Challenge on learned image compression. https://www.tensorflow.org/datasets/catalog/clic.

[95] Shannon C.E. (1948). A mathematical theory of communication. Bell Syst. Tech. J..

[96] Goldsmith A. Joint source/channel coding for wireless channels. Proceedings of the IEEE Vehicular Technology Conference.

[97] Vembu S., Verdu S., Steinberg Y. (1995). The source-channel separation theorem revisited. IEEE Trans. Inf. Theory.

[98] Gündüz D., Qin Z., Aguerri I.E., Dhillon H.S., Yang Z., Yener A., Wong K.K., Chae C.B. (2023). Beyond Transmitting Bits: Context, Semantics, and Task-Oriented Communications. IEEE J. Sel. Areas Commun..

[99] Kurka D.B., Gündüz D. (2021). Bandwidth-agile image transmission with deep joint source-channel coding. IEEE Trans. Wirel. Commun..

[100] Tung T.Y., Gündüz D. (2022). DeepWiVe: Deep-Learning-Aided Wireless Video Transmission. IEEE J. Sel. Areas Commun..

[101] Wang M., Zhang Z., Li J., Ma M., Fan X. (2021). Deep Joint Source-Channel Coding for Multi-Task Network. IEEE Signal Process. Lett..

[102] Yang M., Bian C., Kim H.S. (2022). OFDM-guided Deep Joint Source Channel Coding for Wireless Multipath Fading Channels. IEEE Trans. Cogn. Commun. Netw..

[103] Shao Y., Gunduz D. (2023). Semantic Communications With Discrete-Time Analog Transmission: A PAPR Perspective. IEEE Wirel. Commun. Lett..

[104] Wu H., Shao Y., Mikolajczyk K., Gündüz D. (2022). Channel-Adaptive Wireless Image Transmission With OFDM. IEEE Wirel. Commun. Lett..

[105] Niu X., Wang X., Gündüz D., Bai B., Chen W., Zhou G. (2023). A hybrid wireless image transmission scheme with diffusion. Proceedings of the 2023 IEEE 24th International Workshop on Signal Processing Advances in Wireless Communications (SPAWC).

[106] Kountouris M., Pappas N. (2021). Semantics-empowered communication for networked intelligent systems. IEEE Commun. Mag..

[107] Gündüz D., Chiariotti F., Huang K., Kalør A.E., Kobus S., Popovski P. (2023). Timely and massive communication in 6G: Pragmatics, learning, and inference. IEEE BITS Inf. Theory Mag..

[108] Li Z., Wang Q., Wang Y., Chen T. The Architecture of AI and Communication Integration towards 6G: An O-RAN Evolution. Proceedings of the 30th Annual International Conference on Mobile Computing and Networking.

[109] Cui Q., You X., Wei N., Nan G., Zhang X., Zhang J., Lyu X., Ai M., Tao X., Feng Z. (2024). Overview of AI and Communication for 6G Network: Fundamentals, Challenges, and Future Research Opportunities. arXiv.

[110] Tao M., Zhou Y., Shi Y., Lu J., Cui S., Lu J., Letaief K.B. (2024). Federated Edge Learning for 6G: Foundations, Methodologies, and Applications.

[111] Park J., Ko S.W., Choi J., Kim S.L., Choi J., Bennis M. (2024). Towards semantic MAC protocols for 6G: From protocol learning to language-oriented approaches. IEEE BITS Inf. Theory Mag..

[112] Van Huynh N., Wang J., Du H., Hoang D.T., Niyato D., Nguyen D.N., Kim D.I., Letaief K.B. (2024). Generative AI for physical layer communications: A survey. IEEE Trans. Cogn. Commun. Netw..

[113] Han X., Wu Y., Gao Z., Feng B., Shi Y., Gündüz D., Zhang W. (2025). SCSC: A Novel Standards-Compatible Semantic Communication Framework for Image Transmission. IEEE Trans. Commun..

[114] Tao Z., Guo Y., He G., Huang Y., You X. (2023). Deep learning-based modeling of 5G core control plane for 5G network digital twin. IEEE Trans. Cogn. Commun. Netw..

[115] Zheng J., Du B., Du H., Kang J., Niyato D., Zhang H. (2024). Energy-Efficient Resource Allocation in Generative AI-Aided Secure Semantic Mobile Networks. IEEE Trans. Mob. Comput..

[116] Liu Z., Du H., Huang L., Gao Z., Niyato D. (2024). Joint Model Caching and Resource Allocation in Generative AI-Enabled Wireless Edge Networks. arXiv.

[117] Wang X., Feng L., Zhou F., Li W. (2024). Joint Power Allocation and Reliability Optimization with Generative AI for Wireless Networked Control Systems. Proceedings of the 2024 IEEE/CIC International Conference on Communications in China (ICCC Workshops).

[118] Tolba B., Elsabrouty M., Abdu-Aguye M.G., Gacanin H., Kasem H.M. (2020). Massive MIMO CSI feedback based on generative adversarial network. IEEE Commun. Lett..

[119] Zeng Y., Qiao L., Gao Z., Qin T., Wu Z., Khalaf E., Chen S., Guizani M. (2024). CSI-GPT: Integrating generative pre-trained transformer with federated-tuning to acquire downlink massive MIMO channels. IEEE Trans. Veh. Technol..

[120] Zhao Z., Meng F., Li H., Li X., Zhu G. (2024). Mining Limited Data Sufficiently: A BERT-inspired Approach for CSI Time Series Application in Wireless Communication and Sensing. arXiv.

[121] Balevi E., Doshi A., Jalal A., Dimakis A., Andrews J.G. (2020). High dimensional channel estimation using deep generative networks. IEEE J. Sel. Areas Commun..

[122] Arvinte M., Tamir J.I. (2022). MIMO channel estimation using score-based generative models. IEEE Trans. Wirel. Commun..

[123] Fesl B., Strasser M.B.F., Joham M., Utschick W. (2024). Diffusion-based generative prior for low-complexity MIMO channel estimation. IEEE Wirel. Commun. Lett..

[124] Yang T., Zhang P., Zheng M., Shi Y., Jing L., Huang J., Li N. (2025). WirelessGPT: A Generative Pre-trained Multi-task Learning Framework for Wireless Communication. arXiv.

[125] Xie H., Qin Z., Li G.Y., Juang B.H. (2021). Deep learning enabled semantic communication systems. IEEE Trans. Signal Process..

[126] Erdemir E., Tung T.Y., Dragotti P.L., Gunduz D. (2022). Generative Joint Source-Channel Coding for Semantic Image Transmission. arXiv.

[127] Zhang G., Li H., Cai Y., Hu Q., Yu G., Qin Z. (2025). Progressive Learned Image Transmission for Semantic Communication Using Hierarchical VAE. IEEE Trans. Cogn. Commun. Netw..

[128] Zhang M., Wu H., Zhu G., Jin R., Chen X., Gündüz D. (2025). Semantics-Guided Diffusion for Deep Joint Source-Channel Coding in Wireless Image Transmission. arXiv.

[129] Zhang H., Tao M. (2025). SNR-EQ-JSCC: Joint Source-Channel Coding with SNR-Based Embedding and Query. IEEE Wirel. Commun. Lett..

[130] Li B., Liu Y., Niu X., Bait B., Han W., Deng L., Gunduz D. (2024). Extreme Video Compression with Prediction Using Pre-trained Diffusion Models. Proceedings of the 2024 16th International Conference on Wireless Communications and Signal Processing (WCSP).

[131] Yilmaz S.F., Niu X., Bai B., Han W., Deng L., Gündüz D. (2024). High perceptual quality wireless image delivery with denoising diffusion models. Proceedings of the IEEE INFOCOM 2024-IEEE Conference on Computer Communications Workshops (INFOCOM WKSHPS).

[132] Guo L., Chen W., Sun Y., Ai B., Pappas N., Quek T. (2024). Diffusion-Driven Semantic Communication for Generative Models with Bandwidth Constraints. arXiv.

[133] Pei J., Feng C., Wang P., Tabassum H., Shi D. (2025). Latent Diffusion Model-Enabled Low-Latency Semantic Communication in the Presence of Semantic Ambiguities and Wireless Channel Noises. IEEE Trans. Wirel. Commun..

[134] Tung T.Y., Kurka D.B., Jankowski M., Gündüz D. (2022). DeepJSCC-Q: Constellation Constrained Deep Joint Source-Channel Coding. IEEE J. Sel. Areas Inf. Theory.

[135] Kurka D.B., Gündüz D. (2020). DeepJSCC-f: Deep joint source-channel coding of images with feedback. IEEE J. Sel. Areas Inf. Theory.

[136] Geng Y., Niu X., Bai B., Han W. (2024). Capacity Bounds of Broadcast Channel with a Full-Duplex Base-User Pair. Proceedings of the 2024 IEEE Information Theory Workshop (ITW).

[137] Jiao T., Ye C., Huang Y., Feng Y., Xiao Z., Xu Y., He D., Guan Y., Yang B., Chang J. (2024). 6G-Oriented CSI-Based Multi-Modal Pre-Ttaining and Downstream Task Adaptation Paradigm. Proceedings of the 2024 IEEE International Conference on Communications Workshops (ICC Workshops).

[138] Delfani E., Pappas N. (2024). Optimizing Information Freshness in Constrained IoT Systems: A Token-Based Approach. IEEE Trans. Commun..

[139] Li J., Zhang W. (2024). Asymptotically Optimal Joint Sampling and Compression for Timely Status Updates: Age-Distortion Tradeoff. IEEE Trans. Veh. Technol..

[140] Qiao L., Mashhadi M.B., Gao Z., Gündüz D. (2025). Token-Domain Multiple Access: Exploiting Semantic Orthogonality for Collision Mitigation. arXiv.

[141] Bachmann R., Allardice J., Mizrahi D., Fini E., Kar O.F., Amirloo E., El-Nouby A., Zamir A., Dehghan A. (2025). FlexTok: Resampling Images into 1D Token Sequences of Flexible Length. arXiv.

[142] Sargent K., Hsu K., Johnson J., Fei-Fei L., Wu J. (2025). Flow to the Mode: Mode-Seeking Diffusion Autoencoders for State-of-the-Art Image Tokenization. arXiv.

[143] Yu L., Lezama J., Gundavarapu N.B., Versari L., Sohn K., Minnen D., Cheng Y., Gupta A., Gu X., Hauptmann A.G. Language Model Beats Diffusion-Tokenizer is key to visual generation. Proceedings of the International Conference on Learning Representations.

[144] Yang W., Du H., Liew Z.Q., Lim W.Y.B., Xiong Z., Niyato D., Chi X., Shen X., Miao C. (2022). Semantic communications for future internet: Fundamentals, applications, and challenges. IEEE Commun. Surv. Tutorials.

[145] Luo X., Chen H.H., Guo Q. (2022). Semantic communications: Overview, open issues, and future research directions. IEEE Wirel. Commun..

[146] Guo S., Wang Y., Zhang N., Su Z., Luan T.H., Tian Z., Shen X. (2024). A survey on semantic communication networks: Architecture, security, and privacy. IEEE Commun. Surv. Tutor..

[147] Chaccour C., Saad W., Debbah M., Han Z., Poor H.V. (2024). Less data, more knowledge: Building next generation semantic communication networks. IEEE Commun. Surv. Tutor..

[148] Wu H., Chen G., Gunduz D. Actions Speak Louder Than Words: Rate-Reward Trade-off in Markov Decision Processes. Proceedings of the The Thirteenth International Conference on Learning Representations.

[149] Hamdi Y., Niu X., Bai B., Gunduz D. Non-interactive Remote Coordination. Proceedings of the Workshop on Machine Learning and Compression, NeurIPS.

[150] Zhang G., Yue Y., Li Z., Yun S., Wan G., Wang K., Cheng D., Yu J.X., Chen T. Cut the crap: An economical communication pipeline for LLM-based multi-agent systems. Proceedings of the International Conference on Learning Representations.

